# Effect of magnesium added to local anesthetics for caudal anesthesia on postoperative pain in pediatric surgical patients: A systematic review and meta-analysis with Trial Sequential Analysis

**DOI:** 10.1371/journal.pone.0190354

**Published:** 2018-01-02

**Authors:** Hiromasa Kawakami, Takahiro Mihara, Nobuhito Nakamura, Koui Ka, Takahisa Goto

**Affiliations:** 1 Department of Anesthesiology, Kanagawa Children’s Medical Center, Yokohama, Kanagawa, Japan; 2 Department of Anesthesiology and Critical Care Medicine, Yokohama City University Graduate School of Medicine, Yokohama, Kanagawa, Japan; Copenhagen University Hospital, DENMARK

## Abstract

**Background:**

Magnesium has been investigated as an adjuvant for neuraxial anesthesia, but the effect of caudal magnesium on postoperative pain is inconsistent. The aim of this systematic review and meta-analysis was to evaluate the analgesic effect of caudal magnesium.

**Methods:**

We searched six databases, including trial registration sites. Randomized clinical trials reporting the effect of caudal magnesium on postoperative pain after general anesthesia were eligible. The risk ratio for use of rescue analgesics after surgery was combined using a random-effects model. We also assessed adverse events. The *I*^2^ statistic was used to assess heterogeneity. We assessed risk of bias with Cochrane domains. We controlled type I and II errors due to sparse data and repetitive testing with Trial Sequential Analysis. We assessed the quality of evidence with GRADE.

**Results:**

Four randomized controlled trials (247 patients) evaluated the need for rescue analgesics. In all four trials, 50 mg of magnesium was administered with caudal ropivacaine. The results suggested that the need for rescue analgesia was reduced significantly by caudal magnesium administration (risk ratio 0.45; 95% confidence interval 0.24–0.86). There was considerable heterogeneity as indicated by an *I*^2^ value of 62.5%. The Trial Sequential Analysis-adjusted confidence interval was 0.04–5.55, indicating that further trials are required. The quality of evidence was very low. The rate of adverse events was comparable between treatment groups.

**Conclusion:**

Caudal magnesium may reduce the need for rescue analgesia after surgery, but further randomized clinical trials with a low risk of bias and a low risk of random errors are necessary to assess the effect of caudal magnesium on postoperative pain and adverse events.

**Trial registration:**

University Hospital Medical Information Network Clinical Trials Registry UMIN000025344.

## Introduction

Caudal anesthesia has been widely used in pediatric anesthesia. It can be administered rapidly and with a high success rate, but the duration of analgesia is often unsatisfactory. To overcome the short duration of analgesic effect of local anesthetics, many types of adjuvants have been investigated.

Activation of the N-methyl-D-aspartate (NMDA) receptor has been reported to have an important role in the process of central sensitization to pain [1]. NMDA antagonists have been investigated for their ability to decrease acute postoperative pain [2]. Ketamine is one of the NMDA antagonists and has been demonstrated to be effective in reducing postoperative pain after caudal administration [3], but an animal study found that intrathecal ketamine has potentially neurotoxic properties [4], so its clinical use in pediatric patients remains limited.

Magnesium is a voltage-dependent calcium ion channel blocker and also a non-competitive NMDA receptor antagonist. A review article collating animal and human trials concluded that magnesium is useful for reducing postoperative pain by enhancing opioid analgesia [5]. Systemic magnesium was demonstrated to be effective in reducing postoperative pain in adult [6] but not in pediatric patients [7]. The reason for this discrepancy is not clear. Neuraxial administration of magnesium is thought to be more effective than systemic administration, since magnesium does not cross the blood-brain barrier [8]. Epidurally administered magnesium in adults has been shown to prolong the time to the first analgesic request by 109 min without delaying the regression of sensory blockade [9], but in pediatric surgical patients, the analgesic effect of caudal administration of magnesium is inconsistent. The aim of this systematic review and meta-analysis was to evaluate whether pediatric surgical patients who receive caudal magnesium have less postoperative pain than those who receive placebo.

## Materials and methods

This study is a systematic review with meta-analysis and Trial Sequential Analysis (TSA). We followed the recommendations of the Preferred Reporting Items for Systematic Reviews and Meta-Analyses (PRISMA) statement [10] and the Cochrane Handbook [11]. Our study protocol and analysis methods were pre-specified and are registered in the University Hospital Medical Information Network (UMIN) Clinical Trials Registry (UMIN000025344, https://upload.umin.ac.jp/cgi-open-bin/ctr_e/ctr_view.cgi?recptno=R000029145, S1 Text).

### Search strategy

The MEDLINE, Cochrane Register of Controlled Trials, EMBASE, and Web of Science literature databases from inception to November 17, 2017 were searched without language restrictions. The reference lists of the retrieved full articles were also searched. In addition, we conducted a search on the clinicaltrials.gov website and in the UMIN Clinical Trials Registry on November 17, 2017. The PubMed search strategy is provided in the S2 Text.

Two authors (HK, NN) independently examined the titles and abstracts of reports identified by the search strategies described above to exclude irrelevant articles. The complete article was retrieved if eligibility could not be determined from the title or abstract. Potentially relevant studies, chosen by at least one author, were retrieved and full-text versions were evaluated. Articles that met the inclusion criteria were assessed separately by each of the two authors, and any discrepancies were resolved by discussion.

We searched for randomized clinical trials that tested the effect of caudally administered magnesium added to local anesthetics compared with no magnesium or placebo on postoperative pain in pediatric patients. We excluded studies in which patients did not receive caudal anesthesia. Studies that did not investigate postoperative pain were also excluded, along with studies in which the subjects were not surgical patients. We excluded non-pediatric patients (aged older than 18 years). We also excluded data from case reports, comments or letters to the editor, reviews, and animal studies.

### Primary and secondary outcomes

The primary outcomes in this meta-analysis were duration of analgesia and need for rescue analgesia. The secondary outcomes were postoperative pain scores and adverse events.

### Collection of data

A data collection sheet was created and included information on: (i) number of patients in the study, (ii) patient age, (iii) American Society of Anesthesiologists (ASA) physical status, (iv) type of anesthesia, (v) surgical procedure, (vi) dose of magnesium, (vii) medication given to control group, (viii) dose and type of local anesthetic used for caudal anesthesia, (ix) duration of analgesia, (x) definition of duration of analgesia, (xi) number of patients who received rescue analgesia (acetaminophen), (xii) observation period for pain, (xii) postoperative pain score and scale used, (xiii) duration of motor block, (xiv) time to micturition, and (xiv) adverse events. Two authors (HK, NN) extracted the data independently from the included studies and cross-checked the data. When the data were not available in the literature or more detailed information was necessary, attempts were made to contact the authors.

### Assessment of risk of bias in individual studies

As described in the Cochrane Handbook for Systematic Reviews of Interventions [12], we assessed the risk of bias in sequence generation, concealment of allocation sequence, blinding of patients, blinding of health care providers, blinding of data collectors, blinding of outcome assessors, incomplete outcome data, selective outcome reporting, and other sources of bias. The risk of bias was classified as “low,” “high,” or “unclear.” Two authors (HK, NN) evaluated the risk of bias in each trial. Any differences in evaluation of bias were resolved by discussion and consensus. Trials with one or more domains at high or unclear risk of bias were deemed to be at high or unclear risk of bias overall.

### Assessment of quality of evidence

We graded the quality of evidence of the main outcomes using the Grading of Recommendations Assessment, Development, and Evaluation (GRADE) approach. Judgments of the quality of evidence were based on the presence or absence of the following: risk of bias, inconsistency, indirectness, imprecision of results, and publication bias. The quality of evidence for the main outcomes was graded as very low, low, moderate, or high. We formulated a summary of findings table using GRADEpro GDT [13].

### Statistical analysis

Given the significant heterogeneity in the definitions of duration of analgesia and the pain scales used in the studies, we decided not to combine the results for duration of analgesia and postoperative pain scores, and that need for rescue analgesia alone was appropriate for meta-analysis. Dichotomous data were summarized using the risk ratio (RR) and 95% confidence interval (CI). If the 95% CI included a value of 1, we considered the difference not to be statistically significant. Heterogeneity was quantified using the *I*^2^ statistic. We used the random-effects model (DerSimonian and Laird method [14]) to combine the results. Forest plots were used to graphically represent and evaluate the effects of treatment. Small-study effects were assessed using a funnel plot and an Egger’s regression asymmetry test [15] when the total number of trials included was more than 9. Small-study effects were considered to be positive if the p-value was < 0.1 in the regression asymmetry test. Sensitivity analyses were performed for the primary outcomes according to the risk of bias (low vs. unclear or high). For our primary outcomes, TSA was performed to correct for random errors and repetitive testing of accumulating and sparse data. TSA monitoring boundaries (i.e., monitoring boundaries for meta-analysis) and required information size were quantified, and adjusted CIs were calculated. The risk of type 1 error was maintained at 5% with a power of 90%. An RR of 0.75 for need of rescue analgesia was considered to be clinically significant. If the TSA-adjusted CI included a value of 1, we considered the difference not to be statistically significant. All statistical analyses were performed using the R statistical software package, version 3.3.0 (R Foundation for Statistical Computing, Vienna, Austria). TSA was performed using TSA viewer version 0.9.5.5 β (www.ctu.dk/tsa).

## Results

**Fig 1:**
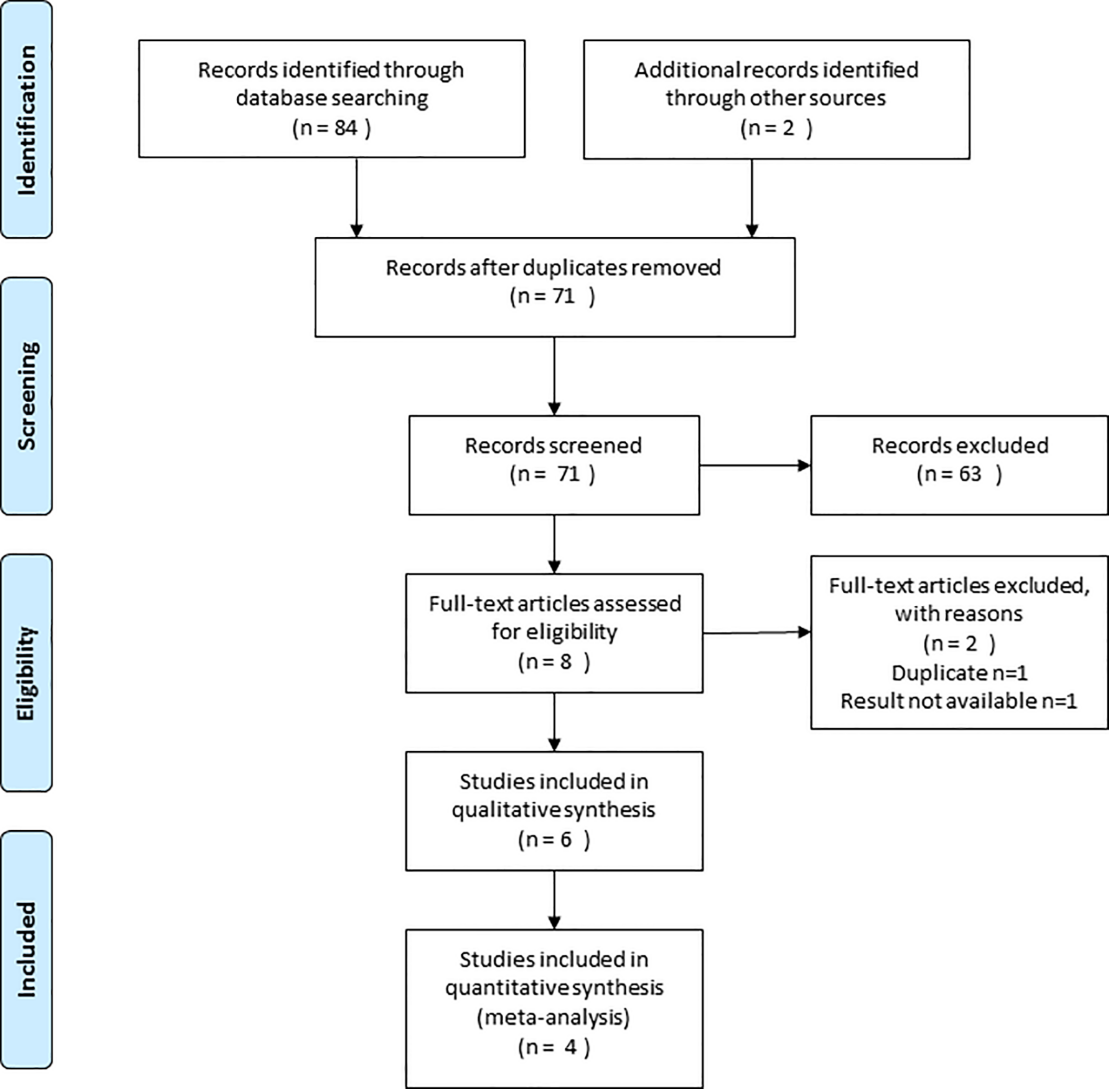
PRISMA flow diagram.

A total of 86 publications were identified, six studies of which could be included in this review. The PRISMA flow diagram detailing the disposition of the retrieved publications is shown in Fig 1. The evaluated trials included data from 371 subjects, 179 of whom received caudal magnesium.

The features of the randomized studies included in this meta-analysis are listed in Table 1. All studies included patients with an American Society of Anesthesiologists physical status of I or II. In the trial by Sridhar et al. [16], the participants underwent infraumbilical surgery with no more details given, while in the rest of the trials, the patients underwent surgery of the male genitalia or the groin. In all trials, participants were predominantly male. The dose of magnesium administered in all of the six trials was 50 mg. Bupivacaine was used for caudal anesthesia in one trial [17] and ropivacaine was used in the rest of the studies. The dose and concentration of ropivacaine varied among the studies.

**Table 1:** Characteristics of included studies.

Reference	Age, years	Patient number (magnesium/ control)	Surgery	Local anesthetics used for caudal anesthesia	Amount of local anesthetics	Scoring tool for pain	Definition of the end of analgesic duration
Birbicer 2007 [18]	2–10	23/37	Inguinal hernia repair, orchidopexy, circumcision	0.25% ropivacaine	0.5 mL/kg	POPS, CHEOPS	Not defined
Elshal 2009 [19]	4–10	20/20	Hypospadias surgery	0.25% ropivacaine	0.5 mL/kg (1 mL/kg in Discussion)	OPS	First dose of analgesia administered (OPS ≥4)
Kim 2014 [20]	2–6	39/38	Inguinal hernia repair	0.15% ropivacaine	1 mL/kg	PPPM	First oral acetaminophen administration after surgery (when PPPM was ≥6)
Yousef 2014 [21]	1–6	35/35	Inguinal hernia repair	0.15% ropivacaine	1.5 mL/kg	CHEOPS, FLACC	Time when FLACC and CHEOP was ≥4
Sridhar 2017 [16]	3–12	32/32	Infraumbilical surgery	0.2% ropivacaine	0.5 mL/kg	MOPS	The time from caudal block to acetaminophen administration (MOPS >4)
Askar 2017 [17]	1–6	30/30	Inguinal orchidopexy, distal hypospadias surgery, inguinal hernia repair	0.25% bupivacaine	1 mL/kg	FLACC	The time from caudal block to the first analgesic administration (FLACC > 7)

POPS, Paediatric Objective Pain Score; CHEOPS, Children’s Hospital of Eastern Ontario Pain Scale; OPS, Objective Pain Scale; PPPM, Parent’s Postoperative Pain Measurement; FLACC, Faces Legs Activity Cry Consolability tool; MOPS, Modified Objective Pain Score

### Duration of analgesia

Five trials reported duration of analgesia. The results for the individual studies are shown in Table 2. The end of the analgesic duration was determined using various pain scales. One trial reported that the duration of analgesia was similar in the two treatment groups, but neither the definition of duration of analgesia nor the duration measured was documented [18]. Our attempts to contact the corresponding author were unsuccessful. Kim et al. [20] only included patients who received rescue acetaminophen if the Parent’s Postoperative Pain Measurement was ≥6 when calculating the duration of analgesia. They reported the median and interquartile range of the time until use of oral rescue analgesia and excluded patients who did not receive oral analgesics from the calculation. Comparing the patients who received rescue analgesia according to treatment group, they concluded that there was no difference in duration of analgesia. Yousef et al. reported the duration of analgesia as the median and 95% CI [21]. The definition of duration of analgesia varied between the studies, and in four studies it was unclear how it was calculated in patients whose pain score did not reach the threshold. Therefore, we decided not to combine the results for duration of analgesia in the meta-analysis. Significant prolongation of analgesic duration was observed in three out of the five trials.

**Table 2:** Duration of analgesia in children who received caudal magnesium in addition to ropivacaine in comparison with that in children who received ropivacaine alone.

	Magnesium group		Control group		
Reference	Patients, n	Duration of analgesia, min	Patients, n	Duration of analgesia, min	p-value
Elshal 2009 [19]	20	222 ± 42^a^	20	192 ± 54^a^	> 0.05
Kim 2014 [20]	39	485 (345–650)^b^	38	390 (360–660)^b^	0.74
Yousef 2014 [21]	35	480 (330–660)^c^	35	240 (180–300)	< 0.001
Sridhar 2017 [16]	32	325 ± 46^a^	32	286 ± 53^a^	< 0.001
Askar 2017 [17]	30	916 ± 103^a^	30	360 ± 139^a^	< 0.001

^a^Mean ± standard deviation.

^b^median (interquartile range).

^c^median (95% confidence interval).

### Need for rescue acetaminophen

**Fig 2:**
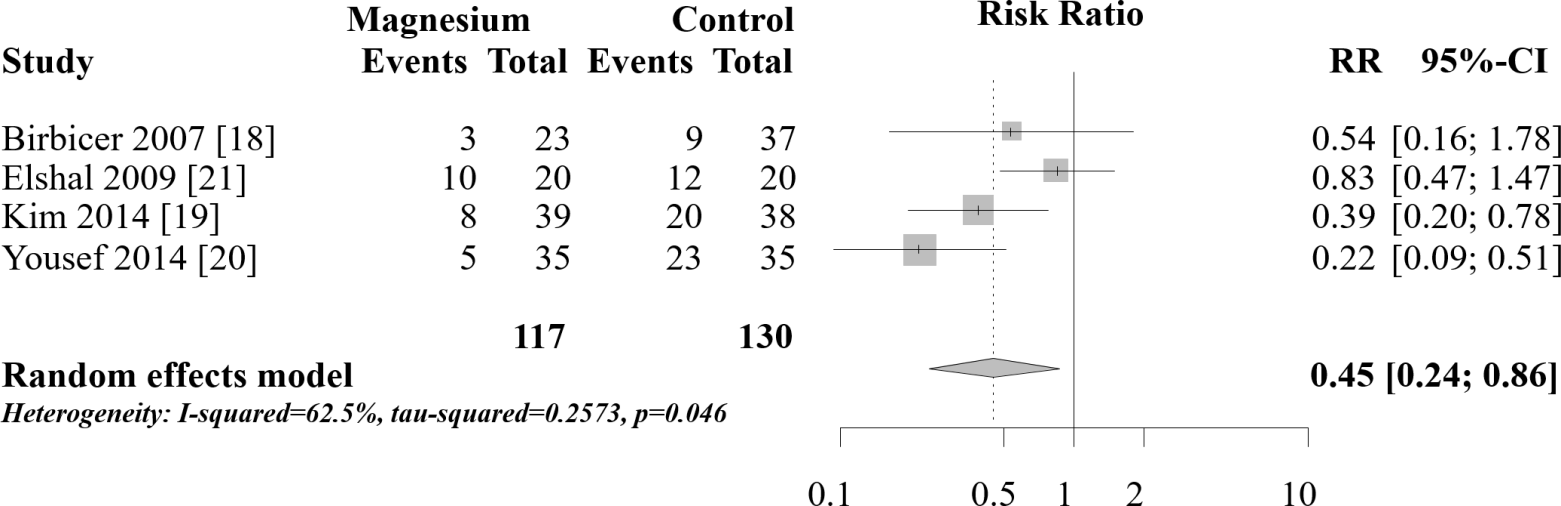
Analysis of the pooled data for numbers of patients requiring postoperative rescue analgesia.

Four trials reported the number of patients who received rescue acetaminophen postoperatively [18–21]. The duration of rescue analgesia in the trials ranged from 6 h [19] to 72 h [20]. The combined results are shown in Fig 2. The need for rescue medication was significantly lower in the magnesium group than in the control group (RR 0.45, 95% CI 0.24–0.86, *I*^2^ statistic 62.5%). Birbicer et al. reported that nine of the 23 patients in their magnesium group and three of 37 patients in their control group received rescue acetaminophen [18]. They reported that this difference was not statistically significant (p>0.05), but a two-tailed chi-squared test of this result indicated significantly worse pain control in the magnesium group (p = 0.0064). If the numbers of patients receiving rescue were opposite, that is, three in the magnesium group instead of nine and nine in the control group instead of three, the p-value would be expected to be >0.05. We attempted to contact the corresponding author but no reply was received. We assume that the authors recorded the numbers incorrectly in the manuscript but included the correct numbers in their analysis. We conducted two sensitivity analyses, one without their trial and another with their trial with the documented results. When the trial is removed from the analysis, the combined result is similar to the original analysis and there is a significant reduction in the rescue use of analgesia (RR 0.43, 95% CI 0.19–0.97). When the study is included with the results as documented in the manuscript, there is no longer a significant difference between the groups (RR 0.71, 95% CI 0.26–1.94).

**Fig 3:**
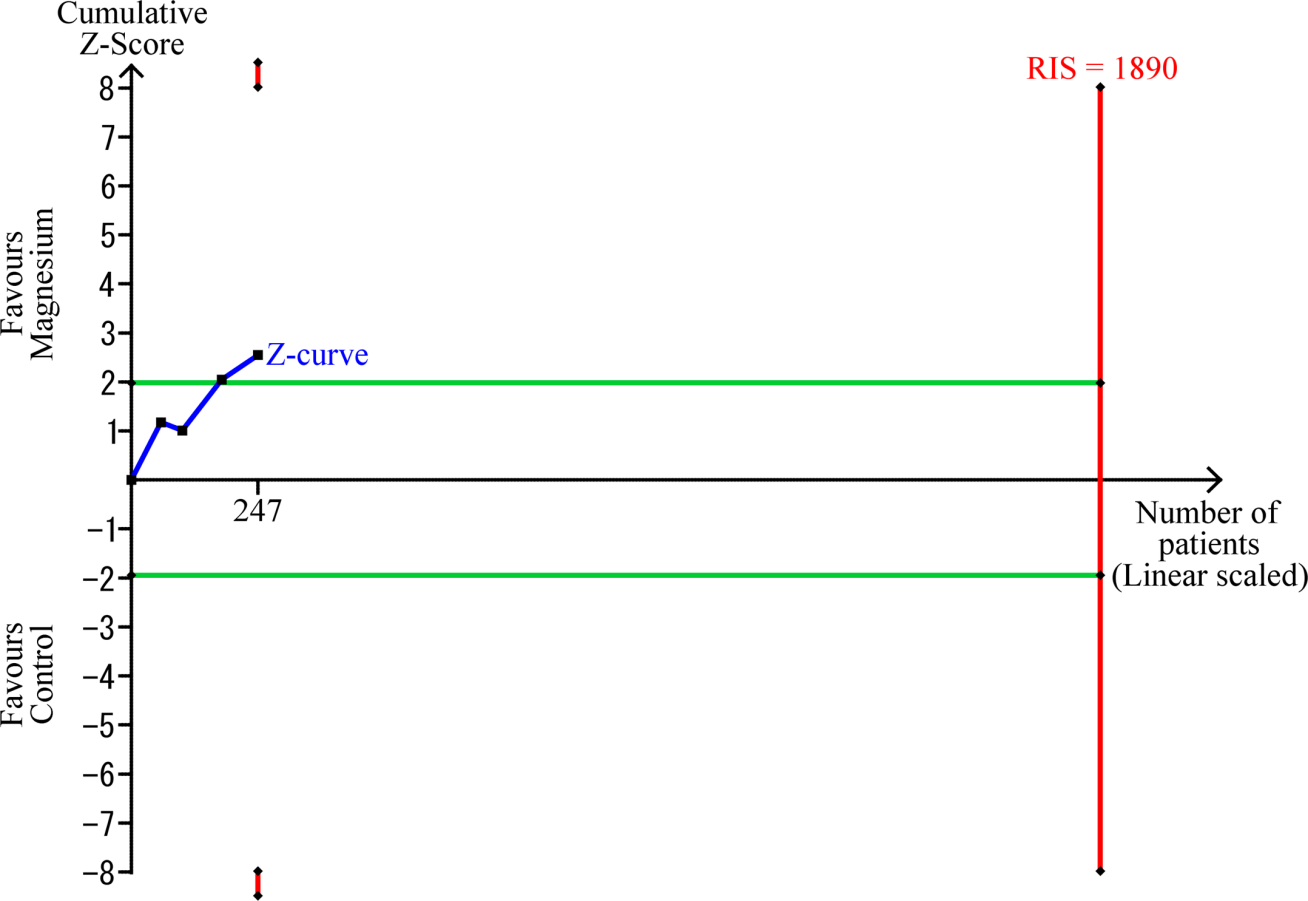
Trial Sequential Analysis for effect of caudal magnesium on numbers of patients requiring rescue analgesia when compared with placebo. The risk of type 1 error was maintained at 5% with a power of 90%. The variance was calculated from the data obtained from the included trials. A clinically significant anticipated relative risk of rescue analgesia was set at 0.75.

The TSA-adjusted CI was 0.04–5.55, indicating that the effect of caudal magnesium on the number of patients requiring rescue analgesia was not statistically significant. The cumulative Z-score did not cross the trial sequential monitoring boundary for benefit (Fig 3). The TSA revealed that the accrued information size (n = 247) reached only 13.1% of the estimated required information size (n = 1890).

## Pain score

The pain score was reported in all six trials. The pain scales used varied between the trials and pain was measured at various time points. Kim et al. reported that the immediate postoperative Faces Legs Activity Cry Consolability (FLACC) score was similar between the groups until 3 h and that the later Parent’s Postoperative Pain Measurement was better in the magnesium group [20]. In one study, the Children’s Hospital of Eastern Ontario Pain Scale and FLACC scores were better in the magnesium group at 4 h but were better in the control group at 8 h [21]. Askar et al. reported that FLACC scores were better in the magnesium group at 2, 4, and 6 h after surgery than in the control group. [17] Sridhar et al. reported that the Modified Objective Pain Score was significantly better at 6 h and 12 h after surgery in the magnesium group [16]. The pain scores were similar in the other two trials. Considering the significant heterogeneity of the pain scales used in the studies, we decided not to combine the pain score results.

### Adverse events

Adverse events were assessed in all six trials. No serious adverse events, such as persistent nerve injury, were reported in any of the six trials. In one trial, it was reported that no adverse events were noted in the postanesthesia care unit (PACU) [16]. Non-serious adverse events such as shivering, sedation, and motor block were reported. In three trials, there was no significant difference in adverse events between the treatment groups [18–20]. Yousef et al. reported that shivering occurred in 20% of patients in their control group and in no patient in their magnesium group (p < 0.05) [21]. Sedation was assessed in four trials, and there was no significant difference between the treatment groups in three trials [16,18,19]. Askar et al. reported that the Ramsay Sedation Score was slightly higher in the magnesium group after 30 and 60 min [17]. Respiratory depression was evaluated in two trials but not found in either of the treatment groups [18,21]. Blood pressure and heart rate were recorded in three trials, and found to be comparable between the groups in two trials [16,19]; in one trial they were slightly lower in the magnesium group for 30 min after admission to the PACU [17]. Time to first micturition was evaluated in two studies and found to be comparable between groups [20,21]. The serum magnesium concentration was measured in one study, and there was no significant change postoperatively from the preoperative measurement in the magnesium group; however, the concentration was not measured in the control group [18].

### Motor block

The level of motor block was assessed using the modified Bromage scale in four trials. Only one trial reported the scores on this scale [21]. In two trials, no motor block was observed [18,20], and motor block levels were reported to be comparable in the other two trials [19,21].

### Risk of bias in the included trials

The risk of bias in the included trials is summarized in Table 3. All six trials were considered to be at unclear risk of bias.

**Table 3:** Risk of bias in the included trials.

	Sequence generation	Allocation concealment	Patients blinded	Health care providers blinded	Data collectors blinded	Outcome assessors blinded	Incomplete outcome data	Selective reporting	Other bias	Summary
Birbicer 2007 [18]	Unclear	Unclear	Low	Low	Unclear	Unclear	Unclear	Low	Low	Unclear
Elshal 2009 [19]	Unclear	Low	Unclear	Low	Low	Low	Low	Unclear	Low	Unclear
Kim 2014 [20]	Low	Unclear	Low	Low	Low	Low	Low	Low	Low	Unclear
Yousef 2014 [21]	Unclear	Unclear	Low	Unclear	Low	Low	Unclear	Low	Low	Unclear
Sridhar 2017 [16]	Unclear	Unclear	Low	Unclear	Unclear	Low	Unclear	Low	Low	Unclear
Askar 2017 [17]	Low	Unclear	Low	Low	Low	Low	Unclear	Low	Low	Unclear

### Sensitivity analysis

A sensitivity analysis according to risk of bias was not performed for the primary outcomes in this meta-analysis, because none of the six trials was at low risk of bias.

### Small study effects

We could not perform an asymmetry test for the funnel plot because only six trials were included in our study.

### Quality of evidence

The quality of evidence for the effect of caudal magnesium on need for rescue acetaminophen as compared with placebo was graded as “very low” (Table 4) because of limitations in study design, inconsistency, imprecision, and possible publication bias. We did not detect indirectness.

**Table 4:** Summary of findings.

**Adding caudal magnesium compared to control for patients with caudal anesthesia**						
**Patients or population**: patients with caudal anesthesia **Intervention**: adding caudal magnesium **Comparison**: control						
Outcomes	Anticipated absolute effects* (95% CI)		Relative effect (95% CI)	Number of participants (studies)	Quality of evidence (GRADE)	Comments
	**Risk in control groups**	**Risk in caudal magnesium groups**				
Patients requiring rescue analgesia	415 per 1,000	**187 per 1,000** (100–357)	**RR 0.45** (0.24–0.86)	247 (4 RCTs)	⨁◯◯◯ Very low^a^^,^^b^^,^^c^^,^^d^	

*The risk in the intervention group (and its 95% confidence interval) is based on the assumed risk in the comparison group and the relative effect of the intervention (and its 95% CI).

CI, confidence interval; RCTs, randomized controlled trials; RR, risk ratio.

^a^All trials were at high risk of bias.

^b^*I*^2^ was >50%.

^c^the 95% CI obtained from the Trial Sequential Analysis was wide.

^d^only four trials were included.

**GRADE Working Group grades of evidence: high quality (w**e are very confident that the true effect lies close to that of the estimate of effect; **moderate quality** (we are moderately confident in the effect estimate; the true effect is likely to be close to the estimate of the effect, but there is a possibility that it is substantially different); l**ow quality** (our confidence in the effect estimate is limited; the true effect may be substantially different from the estimate of the effect); **very low quality** (we have very little confidence in the effect estimate; the true effect is likely to be substantially different from the estimate of effect).

## Discussion

Our meta-analysis included six randomized trials and its results showed that magnesium added to caudal local anesthetics reduced the requirement for rescue analgesia (GRADE, very low). We decided not to combine the results for duration of analgesia, but there was a tendency for this to be longer in the magnesium group in the five studies that evaluated duration of analgesia.

Significantly more patients in the control group received rescue analgesia than those in the magnesium group. Magnesium is a non-competitive NMDA receptor antagonist and has been shown to decrease postoperative pain in adults undergoing certain surgical procedures such as orthopedic and cardiovascular surgeries when administered systemically [6]. Systemic magnesium failed to improve pain after gastrointestinal surgery in adults [6] and after tonsillectomy in a pediatric population [7]. Magnesium has a limited ability to cross the blood-brain barrier [8,22], so intrathecal or epidural administration may be more effective. In addition, systemic magnesium may cause unwanted complications such as prolonged muscle relaxation. NMDA receptors at the dorsal horn are responsible for central pain hypersensitivity [23], and neuraxial magnesium is thought to prevent central sensitization by blocking these receptors. A meta-analysis by Albrecht et al. [9] demonstrated that epidural administration of magnesium increased the time to first rescue analgesic request by 109 min in adult patients. Shruthi et al. demonstrated that epidural magnesium increased sensory block of epidurally administered bupivacaine [24]. Morrison et al. reported that intrathecal administration of magnesium increases the time to first analgesic request only when co-administered with an opioid and not when added to local anesthetics alone [25]. In the trials included in our meta-analysis, no patients were given an opioid intraoperatively either intravenously or caudally, but there was a significant reduction in the number of patients requiring rescue analgesia. This suggests that opioids are not involved in the analgesic effect of caudal magnesium.

The results for use of rescue analgesia showed significant heterogeneity, with an *I*^2^ value of 65%. Although we could not identify the exact reason for this heterogeneity, we suspect differences in the volume of drug administered between the studies may be one of the reasons. In two studies that did not find statistically significant results [18,19], only 0.5 mL/kg of caudal ropivacaine was administered, whereas in the other two trials, 1 mL/kg or more was administered and there was a significant difference between the groups. It is possible that when diluted in a larger volume, magnesium could reach a higher level in the spinal cord. We conducted sensitivity analyses, as one trial reported inconsistent results. When this trial was removed from the meta-analysis, significant differences in rescue use remained. When we included the results as described in the trial, which is inconsistent with the statistical analysis reported in the same trial, no significant differences were found. The outcome of our meta-analysis thus changed depending on the findings of this one study, and we therefore consider our results not very robust.

We could not combine the results for duration of analgesia because of significant clinical heterogeneity. In the trials included in our meta-analysis, five reported duration of analgesia [21] or time to first rescue dose of acetaminophen [16–18,20,21]. Not all patients in these studies received rescue acetaminophen, and when it was administered, various schedules and different pain scales were used. Further, Kim et al. excluded patients who did not receive rescue acetaminophen from calculation of duration of analgesia [20]. It was not clearly documented how the patients not receiving rescue pain medication were handled when obtaining the duration of analgesia in the other two trials [19,21]. Therefore, we decided not to combine the duration of analgesia for meta-analysis. A statistically significant increase in duration of analgesia in the magnesium group was seen in three of the five trials, and the other two trials also showed a tendency for a longer duration of analgesia in the magnesium group.

Time to first rescue analgesia, number of patients who received rescue analgesia, and doses of rescue analgesics are commonly measured outcomes when analyzing postoperative pain. Time to first rescue analgesia is the clinically important outcome, but there seems to be a problem when it is used for comparison of procedures that are not very painful and for which many patients do not need rescue analgesia. Censored patients (i.e., those who do not require rescue medication) could introduce bias. Therefore, we consider that an alternative statistical approach, such as Kaplan-Meier curve analysis and hazard ratio calculation, may be more appropriate for analysis of duration of analgesia.

The optimal dose of magnesium could not be determined in our meta-analysis because all patients in our included trials received 50 mg of magnesium. Buvanendran et al. [26] administered 50 mg of magnesium to adult patients intrathecally. Referring to a previous animal study in which a 188-μg dose of intrathecal magnesium had been administered to rats [27], they calculated their dose of magnesium taking into account the differences in body weight and volume of cerebrospinal fluid between rats and humans. It is not clear how the 50-mg dose of intrathecal magnesium calculated for adults was translated into a 50-mg dose of caudal magnesium for children. A study in adults demonstrated that 50 mg of epidural magnesium significantly reduced postoperative pain [24]. Considering the differences in patient size and weight, a smaller dose of magnesium may be sufficient for postoperative pain.

No serious adverse events were reported in the six included studies. The rate of adverse events, including for sedation and urinary retention, was comparable between the groups. In adults, it has been reported that epidural magnesium increased the duration of motor block [28], but caudal magnesium did not increase motor block in the included trials. Fewer patients experienced postoperative shivering in the magnesium group than in the control group. Epidural administration of magnesium was shown to reduce the incidence of shivering in another study [29], and magnesium may be effective in reducing shivering. Even though all six trials prospectively monitored for adverse events, and magnesium was not associated with an increased number of adverse events, the total number of patients receiving caudal magnesium was only 179, so the possibility of rare but serious adverse events cannot be excluded. In an experimental study in a rat model, 0.02 mL of 15% magnesium sulfate was intrathecally administered [30]. After injection, mobility was not different from a control group, but there were significant changes found at histopathological examination. In another rat study, 0.2 mL of 6.3% or 12.6% intrathecal magnesium sulfate did not cause histological changes [31]. In a case report by Najafi et al., 3 mL of 50% magnesium sulfate was inadvertently administered intrathecally to an adult patient, who then developed bradycardia, hypotension, and loss of consciousness [32]. The patient was intubated for three days, but follow-up magnetic resonance imaging, electromyography, and nerve conduction velocity tests were normal. No long-term complications have been reported after large amounts of intrathecal or epidural administration of magnesium. The safety of large doses of intrathecal magnesium has not been established. The risk of dural puncture during caudal anesthesia has been reported to be 0.008% [33] to 0.1% [34], and even in the cases of dural puncture there was no intrathecal drug injection during caudal anesthesia in over 18,000 pediatric surgical patients [33].

Many drugs have been investigated as adjunct analgesics to caudal local anesthetics. These medications include morphine [35], dexmedetomidine [36], clonidine and neostigmine [37]. Caudal morphine is effective but is known to cause delayed respiratory depression [38]. Neostigmine increases the risk of postoperative nausea and vomiting [37]. Dexmedetomidine is effective and safe [36], but magnesium is less expensive than dexmedetomidine.

There are several limitations to this study. First, the quality of evidence for the effect of caudal magnesium on need for postoperative rescue analgesia was very low. We included only four trials and there is a possibility of publication bias. No study was at low risk of bias. The majority of the included trials had an unclear risk of sequence generation and allocation concealment and there was significant heterogeneity. The concentration and dose of ropivacaine administered caudally varied between the studies and this might have influenced the postoperative analgesic effect. Thus, this systematic review should be considered hypothesis-generative. Second, we did not combine the results for duration of analgesia because of significant clinical heterogeneity. Moreover, we noted serious problems in reporting postoperative duration of analgesia.

## Conclusions

Caudal magnesium might be useful for reducing the need for rescue analgesia after surgery, but the quality of evidence in the literature is very low, and caudal administration of magnesium is still considered experimental. Further well-designed studies that include a more clear definition of duration of analgesia are necessary.

## Supporting information

S1 Table PRISMA checklist. (DOC)

S1 Text Registered study protocol. (DOCX)

S2 Text Search strategy for PubMed. (PDF)

S3 Text Registered study protocol translated into Japanese. (DOCX)
