# Supplementary material for: Effect of magnesium added to local anesthetics for caudal anesthesia on postoperative pain in pediatric surgical patients: A systematic review and meta-analysis with Trial Sequential Analysis
Source: PLoS One. 2018 Jan 2;13(1):e0190354. doi: 10.1371/journal.pone.0190354 (PMC5749796; doi:10.1371/journal.pone.0190354)
Supplement: S1 Text — (DOCX) [file pone.0190354.s002.docx]

**Study Protocol**

**Title:**

Effect of magnesium added to local anesthetics in caudal anesthesia on postoperative pain: A meta-analysis with trial sequential analysis

**Purpose:**

Magnesium has NMDA receptor antagonist effect and previous review showed systemic administration decreases postoperative pain in adults but not in children. There are some randomized control trials that evaluated the analgesic effect of magnesium when administered caudally but these studies yielded different results.

**Methods:**

This study is a systematic review and meta-analysis with trial sequential analysis.

We followed the recommendations of the PRISMA statement and Cochrane Handbook.

**Search strategy**

*Databased searched*

MEDLINE, CENTRAL, Embase, and Web of Science; the reference lists of the retrieved full articles are also searched. Further, we conduct a search of clinicaltrials.gov and the UMIN Clinical Trials Registry.

*Date of search*

We plan to search these databases at November 01, 2016.

The following search strategy combining free text and MeSH terms was set up for PubMed:

(caudal[All Fields] OR "caudal block"[All Fields] OR "caudal epidural"[All Fields]) AND ("magnesium"[MeSH Terms] OR "magnesium"[All Fields]) AND (randomized controlled trial[pt] OR controlled clinical trial[pt] OR randomized[tiab] OR placebo[tiab] OR "drug therapy"[Subheading] OR randomly[tiab] OR trial[tiab] OR groups[tiab]) NOT ("animals"[MeSH Terms] NOT "humans"[MeSH Terms])

Two authors independently scan the titles and abstracts of reports identified by the variety of search strategies described above. If eligibility cannot be determined from the title or abstract, the full paper is retrieved. Potentially relevant studies, chosen by at least one author, are retrieved and evaluated in full-text versions. The articles that meet the inclusion criteria are assessed separately by two authors, and any discrepancies are resolved through discussion.

***Inclusion and Exclusion Criteria***

We search for all randomized controlled trials that tested the efficacy of caudally administered magnesium compared with no magnesium in the postoperative pain in pediatric patients (age less than 18). We exclude studies which did not investigate postoperative pain. We also exclude data from case reports, comments or letters to the editor, reviews, and animal studies. Eligibility is not restricted by language, type of surgery, or anesthetic technique.

***Primary and secondary outcomes***

The primary outcome from the present meta-analysis is duration between analgesic duration and the need for rescue analgesic. The secondary outcome are postoperative pain score, duration of motor block, adverse events.

***Data Collection***

A data collection sheet is created and included data on: (i) number of patients in study, (ii) age, (iii) ASA-Physical Status, (iv) type of anesthesia, (v) type of surgery, (vi) dose of magnesium (vii) medication given to control group, (viii) dose and type of local anesthetics used for caudal anesthesia, (ix) analgesic duration, (x) definition of analgesic duration, (xi) number of patients who received rescue analgesics (acetaminophen) (xii) observation period for pain (xii) postoperative pain score (and scale used) (xiii) duration of motor blocks, (xiv) time to micturition, (xiv) adverse events. Values originally provided as percentages are converted back into actual numbers for analysis. If the data were reported only in graphs which indicated percentages or numbers of patients, we measure the lengths of the graphs to obtain the percentages or numbers of patients. Two authors extract the data independently from the studies included and then cross-check the data.

***Assessment of risk of bias in individual studies***

We assess the risk of bias as described by the Cochrane Handbook for Systematic Reviews of Interventions. We assess the risk of bias in sequence generation, allocation sequence concealment, blinding of patients, blinding of health care providers, blinding of data collectors, blinding of outcome assessors, incomplete outcome data, selective outcome reporting, and other bias. Trials with one or more risks of bias domain that was unclear or at high risk of bias were considered to be trials at high risk of bias.

***Assessment of quality of evidence***

We grade the quality of evidence of the main outcomes using the Grading of Recommendations Assessment, Development, and Evaluation (GRADE). Judgments of the quality of evidence are based on the presence or absence of the following variables: limitations in study design, inconsistency, indirectness, imprecision of the results, and publication bias. The quality of evidence for the main outcomes was graded as very low, low, moderate, or high.

**Statistical Analysis**

Continuous data are summarized using mean difference (MD) with a 95% confidence interval (CI). If the 95% CI include a value of 0, we consider the difference not to be statistically significant. Heterogeneity is quantified with the I^2^ statistic. We use the random effect model (Dersimonian and Laird method) to combine the results. Forest plots are used to graphically represent and evaluate the effects of treatment. Small study effects is assessed using a funnel plot and an Egger’s regression asymmetry test and is considered to be positive if p < 0.1 in the regression asymmetry test. Sensitivity analyses are performed for the primary outcomes according to the risk of bias (low vs. high). For our primary outcomes, trial sequential analysis (TSA) are performed to correct for random error and repetitive testing of accumulating and sparse data. TSA monitoring boundaries (i.e., monitoring boundaries for meta-analysis) and required information size (RIS) are quantified, and adjusted CIs are calculated. Risk of type 1 error is maintained at 5% with a power of 90%. The difference of analgesic duration of 3 hours was considered clinically meaningful. If the TSA-adjusted CI included a value of 0, we consider the difference not statistically significant.

***Software used for statistical analysis***

Statistical analyses are performed using the R statistical software package, version 3.3.0 (R Foundation for Statistical Computing, Vienna, Austria). TSA is performed using TSA viewer version 0.9.5.5 β (www.ctu.dk/tsa).
