# Supplementary material for: Effect of magnesium added to local anesthetics for caudal anesthesia on postoperative pain in pediatric surgical patients: A systematic review and meta-analysis with Trial Sequential Analysis
Source: PLoS One. 2018 Jan 2;13(1):e0190354. doi: 10.1371/journal.pone.0190354 (PMC5749796; doi:10.1371/journal.pone.0190354)
Supplement: S2 Text — (PDF) [file pone.0190354.s003.pdf]

The following search strategy combining free text and MeSH terms was set up for PubMed:

(caudal[All Fields] OR "caudal block"[All Fields] OR "caudal epidural"[All Fields]) AND ("magnesium"[MeSH Terms] OR "magnesium"[All Fields]) AND (randomized controlled trial[pt] OR controlled clinical trial[pt] OR randomized[tiab] OR placebo[tiab] OR "drug therapy"[Subheading] OR randomly[tiab] OR trial[tiab] OR groups[tiab]) NOT ("animals"[MeSH Terms] NOT "humans"[MeSH Terms])
