# Supplementary material for: Effect of magnesium added to local anesthetics for caudal anesthesia on postoperative pain in pediatric surgical patients: A systematic review and meta-analysis with Trial Sequential Analysis
Source: PLoS One. 2018 Jan 2;13(1):e0190354. doi: 10.1371/journal.pone.0190354 (PMC5749796; doi:10.1371/journal.pone.0190354)
Supplement: S3 Text — (DOCX) [file pone.0190354.s004.docx]

**研究プロトコール**

**タイトル:**

仙骨硬膜外に投与する局所麻酔薬にマグネシウムを加えることが術後鎮痛に及ぼす影響： メタアナリシスと試験連続解析(Trial Sequential Analysis)

**目的:**

マグネシウムはNMDA受容体のアンタゴニストであり、過去のレビューで成人において全身投与で術後の鎮痛を減らすと報告されたが小児ではそういった報告はない。仙骨硬膜外に投与したマグネシウムの鎮痛効果について、いくつかの無作為化試験が行われたが、結果は様々であり、はっきりした結論が得られていない。

**方法:**

本研究は、システマティックレビューとメタ解析、試験連続解析である。PRISMA宣言とコクランハンドブックの推奨にしたがって行った。

**検索の方法**

*検索したデータベース*

MEDLINE, CENTRAL, Embase, and Web of Science; 全文を取り寄せた文献の引用文献も検索した。さらに研究登録サイトである clinicaltrials.gov と the UMIN Clinical Trials Registryも検索対象とした。

*検索日*

データベースの検索は 2016年11月1日に行う。

Pubmedにおける検索は以下の検索式を使う。

(caudal[All Fields] OR "caudal block"[All Fields] OR "caudal epidural"[All Fields]) AND ("magnesium"[MeSH Terms] OR "magnesium"[All Fields]) AND (randomized controlled trial[pt] OR controlled clinical trial[pt] OR randomized[tiab] OR placebo[tiab] OR "drug therapy"[Subheading] OR randomly[tiab] OR trial[tiab] OR groups[tiab]) NOT ("animals"[MeSH Terms] NOT "humans"[MeSH Terms])

上の方法で集められた報告を、著者の二人が独立して、タイトルと抄録を見て、本研究の内容に適合するかを検討する。タイトルと抄録だけからでは適合の判断がつかないときには全文を取り寄せて判断する。少なくとも一人の著者が適合と判断する関係する可能性のある研究は、全文を取り寄せる。適合と判断された研究は、二人の著者が独立して評価し、評価が一致しない場合には議論をして結論をだす。

***適合と除外の基準***

小児患者(年齢１８未満)の仙骨硬膜外へのマグネシウムの投与とマグネシウムの投与なしの術後鎮痛への評価を調べた無作為化比較試験を対象とする。述語の鎮痛を評価していない研究は除外する。症例報告･コメント･エディターへのレター･レビュー･動物実験は除外する。言語･術式･麻酔方法の制限はない。

***プライマリアウトカムとセカンダリアウトカム***

プライマリアウトカムは鎮痛の時間とレスキューを必要とする患者の数とするセカンダリアウトカムは術後の疼痛スケール、運動ブロックの時間、合併症とする。

***データの収集***

データ収集用紙を用い、以下のデータを抽出する。 (i) 研究に参加した患者数, (ii) a年齢, (iii) ASA-全身状態, (iv) 麻酔方法, (v) 術式, (vi) マグネシウムの投与量 (vii) 対照群に投与した薬剤, (viii) 仙骨硬膜外に投与した局所麻酔薬の濃度、種類、量, (ix) 鎮痛時間, (x) 鎮痛時間の定義, (xi) アセトアミノフェンのレスキューを受けた患者の数 (xii) 鎮痛の有無を観察した期間 (xii) 術後の疼痛スコア (用いたスケール) (xiii) 運動ブロックの期間, (xiv) 排尿までの時間, (xiv) 合併症。割合で報告された値は解析のためには実際の数に変換する。患者の割合や数でグラフとしてのみ報告されている値がある場合にはグラフ上の長さを計測して値を得る。２人の著者が独立してデータを抽出し、そのデータを相互にチェックする。

***個々の研究のバイアスのリスクの評価***

Cochrane Handbook for Systematic Reviews of Interventionsで示された方法でバイアスのリスクを評価する。我々は、ランダム割り付けの順番、割り付けの隠蔽、患者の盲検化、医療者の盲検化、データ収集者の盲検化、アウトカム評価者の盲検化、不完全なアウトカムへの対処、アウトカムの選択的報告、その他について、バイアスのリスクを「低い」「高い」「不明」のいずれかに分類する。１つ以上の領域でバイアスのリスクが高いまたは不明と評価された研究の全体のバイアスのリスクは高いとする。

***エビデンスの質の評価***

プライマリアウトカムのエビデンスの質はthe Grading of Recommendations Assessment, Development, and Evaluation (GRADE)アプローチを用いて等級分けする。エビデンスの質の判断は、研究デザインの限界・非一貫性・エビデンスの非直接性・データの不精確さ･出版バイアスの５つのエビデンスの質を下げる要因の有無に基づいて行う。エビデンスの質は「非常に低」「低」「中」「高」の４段階に等級分けする。

**統計解析**

連続データは、平均の差と９５％信頼区間を使ってまとめる。95％信頼区間に0が含まれれば、その差は統計学的に有意でないと判断する。二分のデータは相対危険度と95％信頼区間を用いてまとめ、95％信頼区間に1が含まれない場合は統計的に有意な差がないと判断する。異質性はI2を用いて評価する。結果の統合にはランダム効果モデル（Dersimonian and Laird method) を用いる。フォレストプロットを用いて、治療の効果を図示し評価する。小規模研究の影響をファンネルプロットとEggerの回帰非対照試験を用いて評価する。プライマリアウトカムについてバイアスのリスクに応じて感度分析をする。プライマリアウトカムに対して試験連続解析（TSA）をして、データの蓄積と繰り返しの解析によって起こるランダムエラーの補正をする。TSAモニタ境界を計算してRequired information sizeを算出し、補正信頼区間を計算する。タイプ1エラーのリスクを90％として、パワーを90％とする。鎮痛時間の差が3時間あるいはレスキューを必要とする患者数が25％変化すると臨床的に意義がある判断する。

***統計解析のソフトウェアSoftware used for statistical analysis***

統計解析はhe R statistical software package, version 3.3.0 (R Foundation for Statistical Computing, Vienna, Austria)を用いる。TSAはTSA viewer version 0.9.5.5 β ([www.ctu.dk/tsa](http://www.ctu.dk/tsa))を用いて行う
